# Towards a unified and standardized definition of the frailty phenotype: Rumo a uma definição unificada e padronizada do fenótipo de fragilidade

**DOI:** 10.1590/1516-3180.2016.00220162706

**Published:** 2016-09-26

**Authors:** Claudia Kimie Suemoto

**Affiliations:** I MD, PhD. Professor, Discipline of Geriatrics, Faculdade de Medicina da Universidade de São Paulo (FMUSP), São Paulo, SP, Brazil. Brazil

Frailty among older adults has been associated with several adverse health outcomes, including falls, hospitalization, functional decline and death.^1^ Although the prevalence of frailty is increasing, with high morbidity and mortality among older adults, systematic description of the frailty phenotype is recent.^2^ In 2001, Fried et al. defined frailty as a clinical syndrome that is present if three or more of the following criteria are present:^1^ unintentional weight loss of 4.5 kg over the past year, self-reported exhaustion, weakness (measured via grip strength), slow walking speed and low physical activity. The cutoffs for weakness, slowness and low physical activity were defined as the lowest 20^th^ percentile in the Cardiovascular Health Study.^2^

Despite current advances in defining frailty, significant confusion remains, mainly because of multiple definitions for frailty and lack of standardized measurements. In addition to Fried’s definition of the frailty phenotype, other definitions have been proposed. The Frailty Index was developed using data from the Canadian Study of Health and Aging and took into consideration the number of deficits over time (i.e. disability, diseases, physical and cognitive impairments, psychosocial risk factors and geriatric syndromes).^3^ Another popular definition came from the Study of Osteoporotic Fractures (SOF Index), which defined frailty based on the presence of weight loss, inability to rise from a chair without using arms and reduced energy level.^4^

In this issue of the Sao Paulo Medical Journal, two papers address the frailty syndrome using different samples from Brazil. Tavares et al. investigated the association between frailty and cardiovascular risk factors in 205 patients at a tertiary-level hospital.^5^ Using Fried’s definition of frailty, 26% of the sample were frail, 52% were pre-frail and only 22% were non-frail. The cutoffs for weakness and slowness were based on the original description of Fried et al.^2^ In addition, Tavares et al. used the International Physical Activity Questionnaire (IPAQ) to measure physical activity level, while this was defined in accordance with the Minnesota Leisure Time Activities Questionnaire in the original study by Fried et al.^2^ In the study by Tavares et al., the only cardiovascular risk factor associated with frailty was overweight, which was more prevalent among pre-frail patients.

The other paper, which is also published in this issue of the São Paulo Medical Journal, used a sample of community-dwelling older adults who were living in Ribeirão Preto, Brazil (Study of Frailty in Elderly Brazilian Individuals, FIBRA).^6^ Calado et al. found a prevalence of only 9% for frailty, 50% for pre-frailty and 41% non-frailty. The authors used the same criteria as described by Fried et al.,^2^ but they used specific cutoff points for weakness, slowness and low physical activity that were calculated based on the distribution of these variables in the FIBRA study. In addition, they used the Minnesota Leisure Time Activities Questionnaire to measure physical activity, following the original description by Fried et al.^2^ Calado et al.^6^ found baseline associations of frailty with stroke, diabetes, neoplasia, osteoporosis, urinary and fecal incontinence, more medical visits and hospitalizations.

The striking differences in frailty prevalence and related risk factors were probably due to the different characteristics of the samples, given that Tavares et al. used inpatients at a tertiary-level hospital and Calado et al. used community-dwelling older adults. However, the differences in the criteria used to define frailty should also be taken into consideration, as pointed out above. In fact, in a systematic review, the prevalence of frailty among community-dwelling older adults was found to vary enormously, ranging from 4 to 59%.^7^ According to the authors of this review, differences in putting the frailty phenotype into practice were the main reason for the wide differences in prevalence among the studies. Further attempts should be made to unify and standardize the definitions of frailty in order to have a well-defined intervention that could be clearly targeted in clinical randomized trials, with the aim of decreasing the adverse health outcomes relating to frailty.
